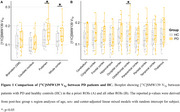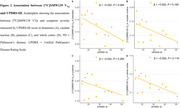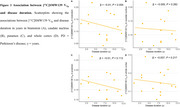# Imaging pro‐inflammatory microglia in Parkinson’s disease using [11C]SMW139 PET: a multi‐center study

**DOI:** 10.1002/alz70861_108572

**Published:** 2025-12-23

**Authors:** Roos M. Rikken, Elsmarieke van de Giessen, Joachim Brumberg, Richard Aarnio, Merijn Joling, Anton Forsberg Morén, Vera Kerstens, Mohammad M Moein, Sangram Nag, Christer Halldin, Patrik Fazio, Dareia S Roos, Henk W. Berendse, Michael Kassiou, Saara Wahlroos, Merja Haaparanta‐Solin, Vesa Oikonen, Robert C. Schuit, Ronald Boellaard, Albert D. Windhorst, Andreas H Jacobs, Adriaan A. Lammertsma, Juha O. Rinne, Andrea Varrone, Sandeep S.V. Golla

**Affiliations:** ^1^ Amsterdam Neuroscience, Brain Imaging, Amsterdam Netherlands; ^2^ Alzheimer Center Amsterdam, Neurology, Vrije Universiteit Amsterdam, Amsterdam UMC location VUmc, Amsterdam Netherlands; ^3^ Radiology & Nuclear Medicine, Vrije Universiteit Amsterdam, Amsterdam UMC location VUmc, Amsterdam Netherlands; ^4^ Department of Radiology and Nuclear Medicine, Vrije Universiteit Amsterdam, Amsterdam University Medical Center, location VUmc, Amsterdam Netherlands; ^5^ Medical Center ‐ University of Freiburg, Faculty of Medicine, University of Freiburg, Freiburg, Baden‐Wuerttemberg Germany; ^6^ Turku PET Centre, Turku University Hospital and University of Turku, Turku Finland; ^7^ Radiology & Nuclear Medicine, Amsterdam UMC location VUmc, Amsterdan Netherlands; ^8^ Karolinska Institutet, Stockholm Sweden; ^9^ Department of Clinical Neuroscience, Centre for Psychiatry Research, Karolinska Institutet and Stockholm Healthcare Services, Stockholm Sweden; ^10^ Clinical Neuroscience, Centre for Psychiatry Research, Karolinska Institutet and Stockholm Healthcare Services, Stockholm Sweden; ^11^ Neurology, Amsterdam UMC location VUmc, Amsterdam Sweden; ^12^ Department of Neurology, Neuroscience Amsterdam, VU University Medical Center, Amsterdam Netherlands; ^13^ School of Chemistry; University of Sydney, Sydney Australia; ^14^ Turku PET Centre University of Turku, Turku Finland; ^15^ Department of Radiology & Nuclear Medicine, Amsterdam Neuroscience, Vrije Universiteit Amsterdam, Amsterdam UMC, Amsterdam Netherlands; ^16^ European Institute for Molecular Imaging, Munster Germany; ^17^ Nuclear Medicine and Molecular Imaging, University of Groningen, University Medical Center Groningen, Groningen Netherlands; ^18^ Turku University Hospital, Turku Finland

## Abstract

**Background:**

Several TSPO PET studies have shown increased glial cell density in Parkinson’s disease (PD). However, TSPO PET cannot discriminate between pro‐ and anti‐inflammatory microglia, thereby limiting the understanding of pro‐inflammatory contributions to PD pathogenesis. Therefore, this study focused on the novel radiotracer [^11^C]SMW139 targeting the P2X7 receptor (P2X7R), which is specifically expressed on pro‐inflammatory microglia. We aim to investigate pro‐inflammatory signals in PD using [11C]SMW139 PET.

**Method:**

15 PD patients (age: 67, 67% male) and 15 controls (HC) (age: 64, 47% male) were included from Amsterdam UMC, Turku University Hospital and Karolinska Institutet. All participants underwent a 90 min [^11^C]SMW139 PET scan with arterial sampling, resulting in the outcome measure V_Tp_ (Volume of distribution). [^11^C]SMW139 V_Tp_ was quantified in several regions of interest (ROIs)(Figure 1). Differences in [^11^C]SMW139 V_Tp_ between PD and HC were assessed using linear mixed models with post hoc testing. Associations between motor symptom severity as measured by UPDRS‐III, disease duration and [^11^C]SMW139 V_Tp_ were assessed using linear regressions.

**Result:**

In the a‐priori ROIs, PD patients showed significantly higher V_Tp_ in the putamen (β=0.04, *p* =0.046) and whole cortex (β= 0.04, *p* =0.043) compared to HC, indicating higher pro‐inflammatory activity (Figure 1). In an exploratory analysis, PD patients also showed higher V_Tp_ in the orbitofrontal cortex (β= 0.04, *p* =0.041). There was no significant association between V_Tp_ and symptom severity (brainstem: β=‐0.002, *p* =0.084; caudate nucleus; β= ‐0.002, *p* = 0.164800; putamen: β= ‐0.002, *p* =0.265, whole cortex: β= ‐0.002, *p* =0.119) or disease duration in PD (brainstem: β= ‐0.01, *p* =0.055; caudate nucleus; β= ‐0.005, *p* = 0.282; putamen: β= ‐0.01, *p* =0.113, whole cortex: β= ‐0.007, *p* =0.217) (Figure 2). However, there was a negative trend indicating higher pro‐inflammatory activation earlier in the disease trajectory, which warrants further investigation (Figure 2 & 3).

**Conclusion:**

PD patients showed increased P2X7R binding in the putamen and brain cortex, as assessed by [^11^C]SMW139 PET, suggesting the presence of increased levels of pro‐inflammatory microglia.